# Tuberculous meningitis: presentation, diagnostic and outcome in HIV-infected individuals from regional center Iaşi

**DOI:** 10.1186/1471-2334-13-S1-P2

**Published:** 2013-12-16

**Authors:** Andra Teodor, Liviu Jany Prisăcariu, Carmen Manciuc, Cristina Nicolau, Gabriela Jugănariu, Dănuț Teodor, Carmen Dorobăț

**Affiliations:** 1 “Gr.T.Popa” University of Medicine and Pharmacy, Iaşi, Romania; 2 Infectious Diseases Hospital “Sf Parascheva”, Iaşi, Romania

## Background

Tuberculous meningitis (TBM) is a growing problem in HIV-infected patients. Our objectives were to analyze the main clinical and laboratory features and immuno-virological particularities and outcomes of HIV-infected individuals with TBM.

## Methods

We performed a retrospective study of HIV-infected patients admitted for TBM to the Hospital of Infectious Diseases Iaşi in the last 10 years.

## Results

We registered 21 cases (median age 23, 47% male), out of which 47% had pulmonary TB previously. Clinical features were: abrupt onset 57%, headache 47%, fever 66%, vomiting 38%, respiratory symptoms 38%, coma 9.5%, cranial nerve palsy 19%, meningeal syndrome 38%, altered level of consciousness 19%. Laboratory findings of cerebrospinal fluid: clear aspect 81%, pleocytosis less than 500/cmm 66%, protein level more than 1 g/L 91%, glucose level less than 0.4 g/L 71%, decreased chlorine level 57%. Radiologically we found adenopathy in the pulmonary hilum in 24% of patients. Neurological complications occurred in 24% and drug related liver toxicity in 33%. We identified 47% of HIV-infected persons during 1987-1990. The interval between the diagnosis of the HIV-infection and the present hospital admission had a median of 6 years. The median value of the actual CD4 count was 92/cmm and viral load 570,000 copies/mL. Prior antiretroviral therapy (ART) was administered in 80% of patients with a median of 4 regimens. Only 19% of the patients were classified as adherent to the ART. The antiretroviral therapy administrated during the present hospital admission was: AZT/3TC + EFV – 9.5%, ABC/3TC + EFV – 9.5%, ABC/3TC + KLT – 4.7%, ABC + RAL + T20 – 4.7%. The median period of hospitalization was 10 days. The overall mortality during hospitalization was 43% and at 9 months it was 29%. The 9-month survival rate was 28%.

## Conclusion

Immunological and virological failure generated by non-adherence favored the occurrence of TBM. Although the CNS penetration-effectiveness score of the current antiretroviral regimen was above 8, this did not influence the mortality rate in neuro-meningeal tuberculous infection. In HIV-associated TBM, the clinical course and dismal outcome are undoubtedly influenced by the profound immunosuppression at presentation, emphasizing the need for earlier diagnosis of the HIV infection and initiation of antiretroviral treatment.

